# Treatment of Acute Ischaemic Stroke and Concomitant Multiple Arterial Splanchnic Thromboses in a Patient with Immune Thrombocytopenia on Thrombopoietin Agonist: A Case Report

**DOI:** 10.3390/neurolint15030074

**Published:** 2023-09-18

**Authors:** Senta Frol, Janja Pretnar Oblak, Mišo Šabovič, Pawel Kermer, Matjaž Sever

**Affiliations:** 1 Department of Vascular Neurology, University Medical Centre Ljubljana, 1000 Ljubljana, Slovenia; 2 Faculty of Medicine, University of Ljubljana, 1000 Ljubljana, Slovenia; 3 Department of Vascular Disorders, University Medical Centre Ljubljana, 1000 Ljubljana, Slovenia; 4 Department of Neurology, Nordwest-Krankenhaus Sanderbusch, Friesland Kliniken GmbH, 26452 Sande, Germany; pkermer@gwdg.de; 5 Department of Neurology, University of Göttingen, Robert-Koch Str. 40, 37075 Göttingen, Germany; 6 Department of Haematology, University Medical Centre Ljubljana, 1000 Ljubljana, Slovenia

**Keywords:** acute ischaemic stroke, immune thrombocytopenia, treatment, case report

## Abstract

Immune thrombocytopenia (ITP) is an autoimmune blood disorder characterised by isolated severe thrombocytopenia. Arterial thrombotic events, such as acute ischaemic stroke (AIS), are rare complications. A 56-year-old woman with chronic ITP on eltrombopag and dexamethasone therapy presented to the emergency department due to AIS in the vertebrobasilar territory, and lower abdominal pain. The computed tomography (CT) scan of the head was unremarkable, whereas CT angiography revealed left vertebral artery occlusion. As the platelet count was sufficient, intravenous thrombolysis (IVT) was initiated. However, after 15 min, an anaphylactic reaction occurred, which was appropriately solved. Although the IVT was prematurely stopped, the NIHSS score improved from 7 to 2, and the follow-up head CT scan remained unremarkable. CT angiography of the thoracoabdominal aorta revealed multiple thrombi in the infrarenal aorta, inferior mesenteric artery (IMA), and left renal artery. The abdominal pain subsided after IVT, but recurred within 24 h. Repeated CT angiography showed ischaemia of the descending colon, with persistent IMA occlusion. After the hemicolectomy condition stabilised. Discrete left-sided ataxia and impaired sensation were the only neurological sequelae. We found two articles reporting only three patients with ITP who suffered AIS and were treated with IVT. A favourable outcome was observed in two cases, while one patient suffered an intracranial haemorrhage (ICH) and died. A review of AIS cases with undefined thrombocytopenia treated with IVT reported ICH in up to 6.8% of patients. Our case suggests that IVT for AIS may be effective in patients with ITP. Further data are needed to better clarify this issue.

## 1. Introduction

Immune thrombocytopenia (ITP) is an autoimmune blood disorder characterised by isolated severe thrombocytopenia [1]. The condition has an incidence of 2.9/100,000 [2]. The most typical acute presentation of ITP is wet purpura, with fatigue as the most common chronic symptom. The pathophysiology of ITP is complex, and involves B- and T-cell defects leading to the failure of self-tolerance mechanisms and platelet autoimmunity as a consequence. The diagnosis of ITP is confirmed after the exclusion of other secondary causes of thrombocytopenia. The management of ITP is focused on bleeding prevention, with the elevation of platelets above 20–30 × 10^9^/L. The standard approach is immune suppression with methylprednisolone, rituximab, cyclosporine, or mycophenolate, with varying levels of success, and splenectomy as the last resort. In the last 20 years, the development of thrombopoietin (TPO) mimetic agents, such as eltrombobag and immunosuppression, has significantly improved patient management, outcomes, and safety. The success rate of TPO mimetics is reported to be as high as 80%, and patients experience a better quality of life through avoiding the complications of immunosuppression [3].

Haemorrhagic stroke is a known complication in patients with ITP, while thrombotic events such as acute ischaemic stroke (AIS) are rare. If they occur, they are more likely to involve the venous system [4]. Thromboses occur due to the disease itself, during ITP therapy or, rarely, due to the manifestation of a newly diagnosed antiphospholipid syndrome [5].

Intravenous thrombolysis (IVT) is the gold standard for AIS treatment. According to American and European guidelines, significant thrombocytopenia (platelet level < 100 × 10^9^/L) is a relative contraindication for IVT [6,7]. Namely, patients with thrombocytopenia were excluded from randomised clinical trials (RCTs) for IVT due to possible haemorrhagic complications [1]. It should be emphasised that platelet levels of <100 × 10^9^/L determined as a contraindication for IVT were adapted from expert opinions, rather than based on RCT data. Thrombocytopenia is not considered a contraindication for endovascular treatment (EVT), but data on EVT in ITP patients are lacking.

In this manuscript, we present a patient with ITP who suffered AIS and was treated with IVT. We then summarise and discuss the current knowledge on IVT treatment in patients with ITP. Our findings could be useful for stroke physicians in the treatment of AIS in patients with ITP.

## 2. Case Report

A 56-year-old right-handed woman with ITP on eltrombopag 50 mg OD and dexamethasone therapy presented to the emergency department with a sudden onset of vertigo, nausea, severe left-sided ataxia, horizontal nystagmus, and left-sided paresthesias, which had developed two hours earlier. Seven days prior to the event, she was seen by a haematologist due to the worsening of her ITP, which was diagnosed in 2020, with a platelet count of 16 × 10^9^/L. Due to previous relapses and her known therapeutic response, continuous eltrombopag 50 mg OD and dexamethasone 40 mg OD for four consecutive days was prescribed. A past single administration of eltrombopag, months before the stroke event described here, resulted in an adequate platelet response, without complications.

**Figure 1:**
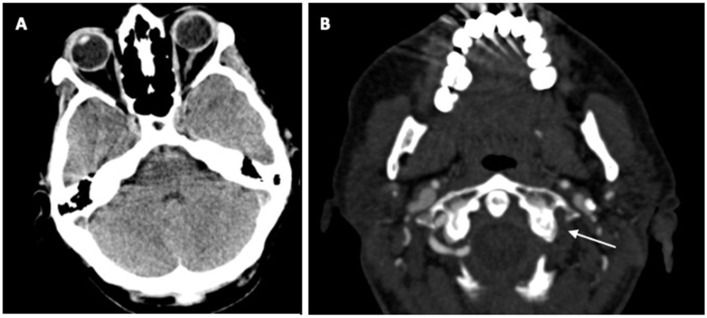
Head CT scans upon admission. (**A**) The native CT scan did not reveal any early signs of ischaemia. (**B**) CT angiography revealed occlusion of the left vertebral artery.

**Figure 2:**
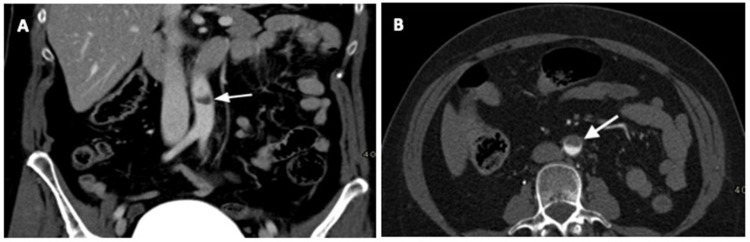
Abdominal CT scans obtained upon admission. (**A**) CT angiography showing a 10 mm long thrombus on the anterior side of the infrarenal aorta. (**B**) The occlusion of the inferior mesenteric artery.

The native computed tomography (CT) and CT perfusion of the head were unremarkable (shown in Figure 1A). CT angiography (CTA) revealed occlusion of the left vertebral artery (VA) in the V4 segment (shown in Figure 1B). Several minutes after urgent imaging diagnostics, her neurological symptoms worsened, with severe paresis of the left arm and dysarthria, resulting in a National Institutes of Stroke Scale (NIHSS) score of 7, and a modified Rankin score (mRs) of 4. The platelet count by the time of AIS had increased to 109 × 10^9^/L. She was reporting lower abdominal pain, with suspicion of further haemorrhagic or thrombotic complications. Therefore, urgent abdominal CT and CTA scans of the abdominal and thoracic aorta were performed. They revealed a thrombus on the anterior side of the infrarenal aorta (shown in Figure 2A), the occlusion of the inferior mesenteric artery (IMA) (shown in Figure 2B), a small thrombus in the left renal artery, an infarct of the lower part of the left kidney, and the hypoperfusion of the lower part of the colon descendens and sigma. The immediate consultation among a haematologist, a vascular neurologist, interventional radiologists, and a urologist resulted in the decision to use IVT, due to significant neurological deficits, a sufficient platelet count >100 × 10^9^/L, and an excellent mRS (0) prior to the stroke. Fifteen minutes after the application of the tissue plasminogen activator (tPA), she developed an anaphylactic reaction with generalised urticaria, itching, and erythroderma. Intravenous thrombolysis (IVT) was stopped immediately, and treatment with intravenous steroids and clemastine was initiated. Although IVT was discontinued, the left arm paresis and dysarthria improved (NIHSS 2), and the abdominal pain resolved. The control head CT scan 24 h after IVT did not reveal ischaemia or haemorrhage. During stroke unit treatment, an electrocardiogram (ECG) revealed a sinus rhythm, and the patient remained haemodynamically stable. The same day, she reported recurrent abdominal pain, and ileus was diagnosed. Repeated abdominal CTA revealed a still-occluded IMA and a smaller thrombus on the anterior side of the infrarenal aorta, while the thrombus in the left renal artery had resolved.

**Figure 3:**
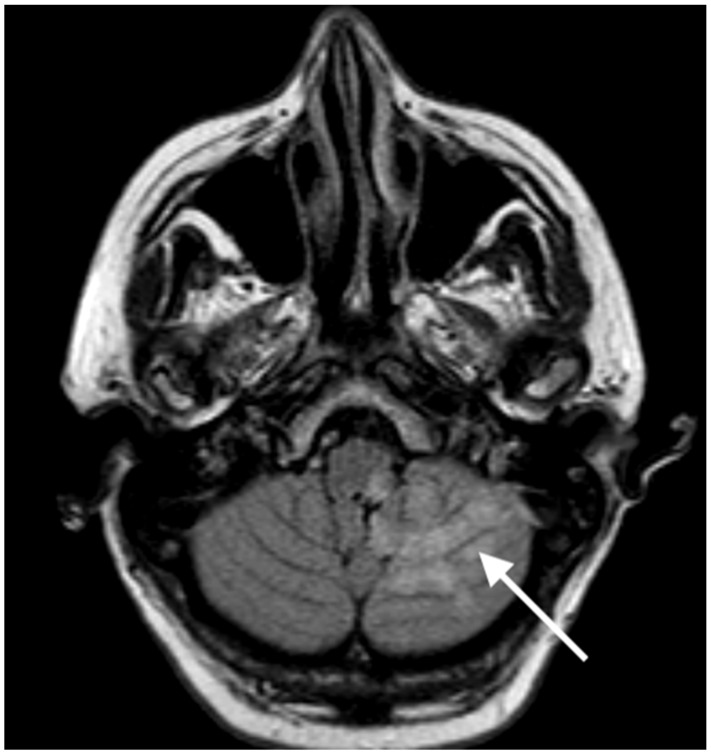
MRI scan obtained 11 days after admission. Image showing an ischaemic stroke in the left dorsolateral medulla oblongata and left cerebellum, in the territory of the left posterior inferior cerebellar artery.

The control abdominal CT scan uncovered ischaemia of the descending colon, resulting in hemicolectomy. The control head CT scan prior to abdominal surgery showed a 3 × 3.5 × 3 cm infarction in the left cerebellum, without haemorrhagic transformation. The neurological status remained unchanged. The platelet count was regularly checked, and remained stable. Following successful surgery, the patient was put on aspirin 100 mg OD, and a prophylactic dose of low-molecular-weight heparin (LMWH) dalteparin 5000 IE subcutaneously. Seven days after IVT, she was transferred back to the neurology department. Mild left-sided ataxia and an impaired sensation of pain and temperature on the left facial side, and contralaterally on the body, persisted (NIHSS 2). Neurorehabilitation was started, and an additional risk factor assessment was performed. A magnetic resonance imaging (MRI) scan of the head revealed an ischaemic stroke in the left dorsolateral medulla oblongata and left cerebellum (the territory of the left posterior inferior cerebellar artery (PICA) (shown in Figure 3). Neurovascular duplex sonography revealed a well-recanalised left VA. Polysomnography did not uncover any central breathing disorders. Echocardiography showed a mild left-ventricular hypertrophy, normal left-ventricular function, and no thrombi inside the cardiac cavities. A Holter ECG revealed a stable sinus rhythm, without paroxysms of atrial fibrillation. A transcranial Doppler ultrasound with the Valsalva maneuver excluded right-to-left shunt. The control CTA of the thoracic and abdominal aorta showed a persisting occlusion of the AMI, the recanalisation of the left renal artery, and the regression of the thrombus in the aorta. An abdominal CT scan showed an infarct of the lower part of the left kidney, and no new pathological changes. Due to the changing and difficult-to-manage values of the repeatedly examined platelet counts (e.g., 50 × 10^9^/L on day 14) eltrombopag 25 mg OD and methylprednisolone in diminishing doses were prescribed, after which the platelet count increased again. Twenty-seven days after being admitted, the patient was discharged to the rehabilitation facility, with very discrete left-sided ataxia, and an impaired sensation of temperature and pain on the left side of the face and contralateral side of the body (NIHSS score of 2). She needed some walking assistance due to ataxia of the left leg (mRS 3). Her medication at discharge consisted of aspirin 100 mg OD, a prophylactic dose of dalteparin subcutaneously, methyprednisolone 32 mg OD, and eltrombopag 25 mg every four days. Regular checks of complete blood count twice weekly, consultations with the haematologist, and follow-up at the outpatient antithrombotic clinic were planned. The last blood count, at the end of January 2023, revealed stable platelet levels of 347 × 10^9^/L, with the methyprednisolone 32 mg planned to be tapered and discontinued. Four months after AIS, the patient’s mRS was 1, with discrete ataxia of the left leg when walking (NIHSS 1).

## 3. Review of the Literature

We searched PubMed up until 15 January 2023 for case reports, case series, trials, reviews, and guidelines reporting treatment of AIS in patients with ITP, using the terms: “acute ischemic stroke” and “immune thrombocytopenic purpura”. In addition, we searched the references of related letters and editorials, to identify other potentially eligible studies. To be eligible for the present analysis, the studies had to be full-text articles published in English language. Duplicates were excluded. This work was reported according to the PRISMA statement [8].

Twenty-four articles were eligible and were included in the review [1,2,5,9,10,11,12,13,14,15,16,17,18,19,20,21,22,23,24,25,26,27,28,29]. We identified three case reports [1,9] and three review articles [1,9,10] on AIS treatment with IVT in patients with ITP. Thirteen articles addressed thrombotic complications in ITP patients [5,11,12,13,14,15,16,17,18,19,20,21,22], and eight articles detailed the thrombotic complications of TPO treatment in ITP patients [2,23,24,25,26,27,28,29].

We identified two articles featuring three ITP patients suffering from AIS and treated with IVT [1,9]. The baseline characteristics and outcomes of these patients, in addition to those of our patient, are presented in Table 1. The mean age of the two male patients was 56 years, with a mean platelet count of 151 × 10^9^/L at AIS presentation. In one patient [9], IVT was discontinued due to a very low platelet count (33 × 10^9^/L). In both patients described by Alrohimi, EVT was performed after IVT, due to large artery occlusion [1]. One patient control CT scan 24 h after reperfusion therapy revealed intracranial haemorrhage (ICH) and subarachnoid haemorrhage [1]. One patient described by Alrohimi et al. died; the other two [1,9] had a very good outcome, with NIHSS 0 at discharge. To our knowledge, there are no published cases of AIS treatment with IVT in ITP patients on eltrombopag.

There are some descriptions of IVT in patients with an unknown cause of thrombocytopenia. In the review by Tomich et al. [9], the authors identified three patients with probable ITP treated with IVT [30,31,32], and none of them had clinical deterioration or suffered symptomatic ICH after IVT [9]. One patient was additionally treated with EVT, with a good clinical outcome [30]. In the review by Alrohimi et al. [1], authors described the outcome of 44 patients with significant thrombocytopenia included in the Thrombolysis in Stroke Patients (TRISP) study [33] who were treated with IVT [1]. Three patients (6.8%) developed symptomatic ICH according to the ECASS-II criteria (Second European Cooperative Acute Stroke Study) [1].

There is also a description of an ITP patient treated with intraarterial thrombolysis. Rhee et al. [5] described a case of intraarterial urokinase treatment due to AIS in an ITP patient with a platelet count of 84 × 10^9^/L at AIS presentation. The treatment outcome was good, without bleeding complications [5].

## 4. Discussion

In this case report, we describe an ITP patient on TPO agonist, presenting with AIS and concurrent multiple splanchnic thromboses/embolisms, whom we treated with IVT. Furthermore, we performed a systematic review of published similar clinical cases.

AIS in ITP patients is a rare event. The literature review and our case report show that the overall outcomes of IVT-treated AIS in ITP patients are good. If the platelet counts prior to IVT are normal, the risk of haemorrhagic complications seems to be 6.8–7.0%. A search of the literature revealed that the rate of haemorrhagic complications was moderately increased, compared to patients without ITP [34,35].

Anaphylactic reactions in patients with AIS are very rare, but patients who received IVT had an 8-fold higher risk of anaphylaxis than patients who did not receive IVT, with the absolute risk of anaphylaxis reaching 1 in 200 patients [36]. There appears to be an independent association between IVT and anaphylaxis in stroke patients. Because alteplase (a tissue plasminogen activator) is an endogenous molecule, it is very unlikely to cause anaphylaxis; it is more plausible that anaphylaxis is a hypersensitivity reaction triggered by the release of vasoactive substances [36]. ITP as an autoimmune disease is not a known cofactor for an anaphylactic reaction, but specific immunotherapy might be [37]. We speculate that the anaphylaxis in our case may be a hypersensitivity reaction to vasoactive substances, which might be exacerbated by immunotherapy for ITP. In cases of systemic or severe reaction, appropriate measures, as performed in our case, and IVT treatment interruption are recommended. As ITP patients with AIS are rare, there are few reports on using alteplase in these patients. We could not identify any other published cases of similar complications. ITP with other autoimmune conditions can develop in children who have atopic diathesis. However, a similar association was not shown to be present in adults [38,39].

Eltrombopag is the standard of care for ITP, usually used as escalation therapy. The success rates are up to 86% for long-term follow-up in patients with chronic or persistent ITP [28]. The incidence of thromboembolic events is an estimated 6%, with the venous system mostly being affected by deep vein thrombosis. The arterial system is most commonly affected, with myocardial infarction and cerebral infarction as atherothrombotic complications that add up to 3% [28]. Practically all arterial events were classified as serious adverse events. The incidence was similar in retrospective and prospective studies with ITP, as well as that reported for romiplostim, another TPO agonist [28]. The recent real-world experience of eltrombopag in ITP reported a similar overall response of 81%, with a thrombosis incidence of 5.6% [29]. Of these, 1.9% were considered SAE, of which one patient died from cerebral venous thrombosis. Several reports of thrombotic complications during eltrombopag treatment were reported, in the context either of ITP [40,41,42,43,44,45] or of secondary thrombocytopenia [46,47,48,49,50,51]. However, our patient represents the only case published to date suffering multiple concurrent thrombotic events.

The start of treatment with eltrombopag is associated with changes in the platelet and coagulation kinetics. TPO agonists not only increase platelet production, but also decrease platelet destruction, leading to a sustained platelet increase in patients [52]. ITP is, by itself, associated with an increased risk of thrombosis. The mechanism is not fully elucidated. In a recent report [53], patients were in a hypercoagulable state, with preactivated platelets, an increased thrombin generation potential, and increased levels of factor VIII and von Willebrand factor. The use of eltrombopag further increased the plasma thrombin generation potential, but not that of other haemostatic parameters [53]. Another approach to looking at changes caused by eltrombopag to haemostasis is to assess microvesicles released from activated/apoptotic cells that are prothrombotic due to exposure to phosphatidylserine and tissue factor, and are increased in ITP patients. TPO agonists increase phospholipid-dependent microvesicle-associated thrombin generation in ITP patients, which could contribute to, or exacerbate, a pre-existing hypercoagulable state [54]. However, the clinical implication of these findings remains to be elucidated.

Our report has limitations. Most importantly, only a few case reports can be found in the literature because of the rarity of the disease and condition. Publication bias, resulting in cases with poor outcomes not being reported, should be considered.

## 5. Conclusions

The treatment of AIS in patients with ITP requires close collaboration between haematology and vascular neurology experts, to find a balance between the benefits and the risks of haemorrhagic complications.

Future stroke studies, such as multicentre prospective cohort studies, that include ITP patients and patients with thrombocytopenia, are needed, to provide evidence-based treatment plans.

## Figures and Tables

**Table 1:** The baseline characteristics and outcomes of patients with definite ITP treated with IVT.

References	Age	Gender	Platelet Count (×10^9^/L)	NIHSS Adm	EVT	Control CT Scan	NIHSS Dis	mRS Dis	mRS 3 Months
Alrohimi et al. [1]	61	F	48	16	1	Ischaemic stroke	0	1	1
	75	M	366	4	1	ICH plus SAH	NA	6	6
Tomich et al. [9]	33	M	80	2	0	NA	0	0	0
Own case report	56	F	109	7	0	Ischaemic stroke	2	3	1
Total data	56	f:m = 1:1	151	7.3	2		NA	2.5	2

Abbreviations: NIHSS, National Institutes of Stroke Scale; EVT, endovascular therapy; CT, computed tomography; ICH, intracerebral haemorrhage; SAH, subarachnoid haemorrhage; mRS, modified Rankin scale; NA, not applicable.

## Data Availability

All the data generated or analysed during this study are included in this article. Further enquiries can be directed to the corresponding author.

## References

[1] Alrohimi A., Purdy K., Alqarni M., Alotaibi G., Blevins G., Butcher K., Rempel J., Wu C., Sun H.L., Khan K. (2021). The Clinical Conundrum of Managing Ischemic Stroke in Patients with Immune Thrombocytopenia. Can. J. Neurol. Sci..

[2] Moulis G., Palmaro A., Montastruc J.L., Godeau B., Lapeyre-Mestre M., Sailler L. (2014). Epidemiology of incident immune thrombocytopenia: A nationwide population-based study in France. Blood.

[3] Provan D., Semple J.W. (2022). Recent advances in the mechanisms and treatment of immune thrombocytopenia. eBioMedicine.

[4] Thachil J., Callaghan T., Martlew V. (2010). Thromboembolic events are not uncommon in 319 patients with immune thrombocytopenia. Br. J. Haematol..

[5] Rhee H.Y., Choi H.Y., Kim S.B., Shin W.C. (2010). Recurrent ischemic stroke in a patient with idiopathic thrombocytopenic purpura. J. Thromb. Thrombolysis.

[6] Powers W.J., Rabinstein A.A., Ackerson T., Adeoye O.M., Bambakidis N.C., Becker K., Biller J., Brown M., Demaerschalk B.M., Hoh B. (2018). 2018 Guidelines for the Early Management of Patients with Acute Ischemic Stroke: A 349 Guideline for Healthcare Professionals from the American Heart Association/American 350 Stroke Association. Stroke.

[7] Berge E., Whiteley W., Audebert H., De Marchis G.M., Fonseca A.C., Padiglioni C., de la Ossa N.P., Strbian D., Tsivgoulis G., Turc G. (2021). European Stroke Organisation (ESO) guidelines on intravenous thrombolysis for acute ischaemic stroke. Eur. Stroke J..

[8] Moher D., Liberati A., Tetzlaff J., Altman D.G., PRISMA Group (2009). Preferred reporting items for systematic reviews and meta-analyses: The PRISMA statement. Ann. Intern. Med..

[9] Tomich C., Debruxelles S., Delmas Y., Sagnier S., Poli M., Olindo S., Renou P., Rouanet F., Sibon I. (2018). Immune-Thrombotic Thrombocytopenic Purpura is a Rare Cause of Ischemic Stroke in Young Adults: Case Reports and Literature Review. J. Stroke Cerebrovasc. Dis..

[10] Mihalov J., Timárová G. (2016). A Seeming Paradox: Ischemic Stroke in the Context of Idiopathic Thrombocytopenic Purpura. Clin. Appl. Thromb. Hemost..

[11] Chandan J.S., Thomas T., Lee S., Marshall T., Willis B., Nirantharakumar K., Gill P. (2018). The association between idiopathic thrombocytopenic purpura and cardiovascular disease: A retrospective cohort study. J. Thromb. Haemost..

[12] Hindi Z., Onteddu N., Ching C.A., Khaled A.A. (2017). Vertebral Artery Thrombosis in Chronic Idiopathic Thrombocytopenic Purpura. Case Rep. Hematol..

[13] Hayashi T., Akioka N., Kashiwazaki D., Kuwayama N., Kuroda S. (2015). Ischemic stroke in pediatric moyamoya disease associated with immune thrombocytopenia—A case report. Childs Nerv. Syst..

[14] Ichijo M., Ishibashi S., Ohkubo T., Nomura S., Sanjo N., Yokota T., Mizusawa H. (2014). Elevated platelet microparticle levels after acute ischemic stroke with concurrent idiopathic thrombocytopenic purpura. J. Stroke Cerebrovasc. Dis..

[15] Theeler B.J., Ney J.P. (2008). A patient with idiopathic thrombocytopenic purpura presenting with an acute ischemic stroke. J. Stroke Cerebrovasc. Dis..

[16] Pan L., Leng H., Huang Y., Xia N., Jin L., Zhang H.T. (2021). Ischemic stroke/transient ischemic attack in adults with primary immune thrombocytopenia: A meta-analysis. Neurol. Sci..

[17] Park H.K., Lee S.H. (2014). Ischemic stroke associated with immune thrombocytopenia: Lesion patterns and characteristics. Neurol. Sci..

[18] De La Peña A., Fareed J., Thethi I., Morales-Vidal S., Schneck M.J., Shafer D. (2012). Ischemic stroke in the setting of chronic immune thrombocytopenia in an elderly patient—A therapeutic dilemma. Clin. Appl. Thromb. Hemost..

[19] Rong X., Jiang L., Qu M., Yang S., Wang K., Jiang L. (2022). Risk factors and characteristics of ischemic stroke in patients with immune thrombocytopenia: A retrospective cohort study. J. Stroke Cerebrovasc. Dis..

[20] Rungjirajittranon T., Owattanapanich W. (2019). A serious thrombotic event in a patient with immune thrombocytopenia requiring intravenous immunoglobulin: A case report. J. Med. Case Rep..

[21] Wang W.-T., Li Y.-Y., Lin W.-C., Chen J.-Y., Lan K.-M., Sun C.-K., Hung K.-C. (2018). Bilateral visual loss and cerebral infarction after spleen embolization in a trauma patient with idiopathic thrombocytopenic purpura: A case report. Medicine.

[22] Zhao H., Lian Y., Zhang H., Xie N., Gao Y., Wang Z., Zhang Y. (2015). Ischemic stroke associated with immune thrombocytopenia. J. Thromb. Thrombolysis.

[23] Rodeghiero F., Stasi R., Giagounidis A., Viallard J.-F., Godeau B., Pabinger I., Cines D., Liebman H., Wang X., Woodard P. (2013). Long-term safety and tolerability of romiplostim in patients with primary immune thrombocytopenia: A pooled analysis of 13 clinical trials. Eur. J. Haematol..

[24] Tjepkema M., Amini S., Schipperus M. (2022). Risk of thrombosis with thrombopoietin receptor agonists for ITP patients: A systematic review and meta-analysis. Crit. Rev. Oncol. Hematol..

[25] Cines D.B., Wasser J., Rodeghiero F., Chong B.H., Steurer M., Provan D., Lyons R., Garcia-Chavez J., Carpenter N., Wang X. (2017). Safety and efficacy of romiplostim in splenectomized and nonsplenectomized patients with primary immune thrombocytopenia. Haematologica.

[26] Palandri F., Rossi E., Bartoletti D., Ferretti A., Ruggeri M., Lucchini E., Carrai V., Barcellini W., Patriarca A., Rivolti E. (2021). Real-world use of thrombopoietin receptor agonists in older patients with primary immune thrombocytopenia. Blood.

[27] Gernsheimer T.B., George J.N., Aledort L.M., Tarantino M.D., Sunkara U., Guo D.M., Nichol J.L. (2010). Evaluation of bleeding and thrombotic events during long-term use of romiplostim in patients with chronic immune thrombocytopenia (ITP). J. Thromb. Haemost..

[28] Wong R.S.M., Saleh M.N., Khelif A., Salama A., Portella M.S.O., Burgess P., Bussel J.B. (2017). Safety and efficacy of long-term treatment of chronic/persistent ITP with eltrombopag: Final results of the EXTEND study. Blood.

[29] Mishra K., Pramanik S., Jandial A., Sahu K.K., Sandal R., Ahuja A., Yanamandra U., Kumar R., Kapoor R., Verma T. (2020). Real-world experience of eltrombopag in immune thrombocytopenia. Am. J. Blood Res..

[30] Idowu M., Reddy P. (2013). Atypical thrombotic thrombocytopenic purpura in a middle-aged woman who presented with a recurrent stroke. Am. J. Hematol..

[31] Sevy A., Doche E., Squarcioni C., Poullin P., Serratrice J., Nicoli F., Weiller P.-J. (2011). Stroke in a young patient treated by alteplase heralding an acquired thrombotic thrombocytopenic purpura. J. Clin. Apher..

[32] Tsai H.M., Shulman K. (2003). Rituximab induces remission of cerebral ischemia caused by thrombotic thrombocytopenic purpura. Eur. J. Haematol..

[33] Gensicke H., Al Sultan A.S., Strbian D., Hametner C., Zinkstok S.M., Moulin S., Bill O., Zini A., Padjen V., Kägi G. (2018). Thrombolysis in Stroke Patients (TRISP) Collaborators. Intravenous thrombolysis and platelet count. Neurology.

[34] Emberson J., Lees K.R., Lyden P., Blackwell L., Albers G., Bluhmki E., Brott T., Cohen G., Davis S., Donnan G. (2014). Effect of treatment delay, age, and stroke severity on the effects of intravenous thrombolysis with alteplase for acute ischaemic stroke: A meta-analysis of individual patient data from randomised trials. Lancet.

[35] Whiteley W.N., Emberson J., Lees K.R., Blackwell L., Albers G., Bluhmki E., Brott T., Cohen G., Davis S., Donnan G. (2016). Risk of intracerebral haemorrhage with alteplase after acute ischaemic stroke: A secondary analysis of an individual patient data meta-analysis. Lancet Neurol..

[36] Lerario M.P., Grotta J.C., Merkler A.E., Omran S.S., Chen M.L., Parikh N.S., Yaghi S., Murthy S., Navi B.B., Kamel H. (2019). Association between intravenous thrombolysis and anaphylaxis among medicare benefeciaries with acute ischemic stroke. Stroke.

[37] Wölbing F., Fischer J., Köberle M., Kaesler S., Biedermann T. (2013). About the role and underlying mechanisms of cofactors in anaphylaxis. Allergy.

[38] Chiang M.R., Wei C.C., Muo C.S., Fu L.S., Li T.C., Kao C.H. (2015). Association of primary immune thrombocytopenia and common allergic diseases among children. Pediatr. Res..

[39] Bahoush G., Poorasgari A., Nojomi M. (2020). Relationship of primary immune thrombocytopenic purpura and atopia among children: A case control study. Sci. Rep..

[40] Diez-Feijóo R., Rodríguez-Sevilla J., Colomo L., Papaleo N., Maiques J., Gimeno E., Andrade-Campos M., Abella E., Merchan B., Calvo X. (2021). Massive intrasplenic arterial thrombosis in a patient with chronic ITP during the development of an Evans syndrome. Thromb. Res..

[41] Saito M., Morioka M., Izumiyama K., Mori A., Kondo T. (2021). Severe Portal Vein Thrombosis During Eltrombopag Treatment Concomitant Splenectomy for Immune Thrombocytopenia. Cureus.

[42] Dichtwald S., Meyer A., Ifrach N. (2021). Catastrophic anti-phospholipid syndrome with Libman-Sacks endocarditis following eltrombopag therapy for immune thrombocytopenic purpura: A case report. Lupus.

[43] Ghumman G.M., Fatima H., Singh G., Khalid T., Ayoubi M. (2023). Risk of Thromboembolism with Eltrombopag: A Case Report of Deep Vein Thrombosis and Bilateral Pulmonary Embolism. Cureus.

[44] Mulla C.M., Rashidi A., Levitov A.B. (2014). Extensive cerebral venous sinus thrombosis following a dose increase in eltrombopag in a patient with idiopathic thrombocytopenic purpura. Platelets.

[45] Gunes H., Kivrak T. (2016). Eltrombopag Induced Thrombosis: A Case with Acute Myocardial Infarction. Curr. Drug Saf..

[46] Iinuma S., Nagasawa Y., Sasaki K., Hayashi K., Kanno K., Honma M., Sugawara M., Kinouchi M., Obata M., Ishida-Yamamoto A. (2020). Cutaneous thrombosis associated with eltrombopag treatment for immune thrombocytopenia. J. Dermatol..

[47] Mohamed S.E., Yassin M.A. (2020). Eltrombopag Use for Treatment of Thrombocytopenia in a Patient with Chronic Liver Disease and Portal Vein Thrombosis: Case Report. Case Rep. Oncol..

[48] Oo Z., Manvar K., Wang J.C. (2022). Eltrombopag-Induced Thrombocytosis and Thrombosis in Patients With Antiphospholipid Syndrome and Immune Thrombocytopenic Purpura. J. Investig. Med. High Impact Case Rep..

[49] Bosi A., Barcellini W., Fattizzo B. (2022). Pulmonary embolism in a patient with eltrombopag-treated aplastic anaemia and paroxysmal nocturnal haemoglobinuria clone during COVID-19 pneumonia. Thromb. J..

[50] Kawano N., Hasuike S., Iwakiri H., Nakamura K., Ozono Y., Kusumoto H., Nagata K., Kikuchi I., Yoshida S., Kuriyama T. (2013). Portal vein thrombosis during eltrombopag treatment for immune thrombocytopenic purpura in a patient with liver cirrhosis due to hepatitis C viral infection. J. Clin. Exp. Hematop..

[51] Baumann A.J., Wheeler D.S., Varadi G., Feyssa E. (2016). Severe Thrombotic Complication of Eltrombopag in a Cirrhotic Patient. ACG Case Rep. J..

[52] Pravdic Z., Suvajdzic-Vukovic N., Djurdjevic P., Pantic N., Bukumiric Z., Virijevic M., Todorovic-Tirnanic M., Thachil J., Mitrovic M. (2023). Platelet kinetics in patients with chronic immune thrombocytopaenia treated with thrombopoietin receptor agonists. Eur. J. Haematol..

[53] Van Dijk W.E.M., Poolen G.C., Huisman A., Koene H.R., Fijnheer R., Thielen N., van Bladel E.R., van Galen K.P.M., Schutgens R.E.G., Urbanus R.T. (2022). Evaluation of the procoagulant state in chronic immune thrombocytopenia before and after eltrombopag treatment-a prospective cohort study. J. Thromb. Haemost..

[54] Garabet L., Ghanima W., Hellum M., Sandset P.M., Bussel J.B., Tran H., Henriksson C.E. (2020). Increased microvesicle-associated thrombin generation in patients with immune thrombocytopenia after initiation of thrombopoietin receptor agonists. Platelets.

